# Searching Novel Therapeutic Targets for Scleroderma: P2X7-Receptor Is Up-regulated and Promotes a Fibrogenic Phenotype in Systemic Sclerosis Fibroblasts

**DOI:** 10.3389/fphar.2017.00638

**Published:** 2017-09-13

**Authors:** Daniela Gentile, Pietro E. Lazzerini, Alessandra Gamberucci, Mariarita Natale, Enrico Selvi, Francesca Vanni, Alessandra Alì, Paolo Taddeucci, Silvia Del-Ry, Manuela Cabiati, Veronica Della-Latta, David J. Abraham, Maria A. Morales, Rosella Fulceri, Franco Laghi-Pasini, Pier L. Capecchi

**Affiliations:** ^1^Department of Medical Sciences, Surgery and Neurosciences, University of Siena Siena, Italy; ^2^Department of Molecular and Developmental Medicine, University of Siena Siena, Italy; ^3^Institute of Clinical Physiology, CNR Pisa, Italy; ^4^Division of Medicine, Department of Inflammation, Centre for Rheumatology and Connective Tissue Diseases, University College London London, United Kingdom

**Keywords:** systemic sclerosis, P2X7 receptor, dermal fibroblasts, collagen, fibrosis, ERK

## Abstract

**Objectives:** Systemic sclerosis (SSc) is a connective tissue disorder presenting fibrosis of the skin and internal organs, for which no effective treatments are currently available. Increasing evidence indicates that the P2X7 receptor (P2X7R), a nucleotide-gated ionotropic channel primarily involved in the inflammatory response, may also have a key role in the development of tissue fibrosis in different body districts. This study was aimed at investigating P2X7R expression and function in promoting a fibrogenic phenotype in dermal fibroblasts from SSc patients, also analyzing putative underlying mechanistic pathways.

**Methods:** Fibroblasts were isolated by skin biopsy from 9 SSc patients and 8 healthy controls. P2X7R expression, and function (cytosolic free Ca^2+^ fluxes, α-smooth muscle actin [α-SMA] expression, cell migration, and collagen release) were studied. Moreover, the role of cytokine (interleukin-1β, interleukin-6) and connective tissue growth factor (CTGF) production, and extracellular signal-regulated kinases (ERK) activation in mediating P2X7R-dependent pro-fibrotic effects in SSc fibroblasts was evaluated.

**Results:** P2X7R expression and Ca^2+^ permeability induced by the selective P2X7R agonist 2′-3′-O-(4-benzoylbenzoyl)ATP (BzATP) were markedly higher in SSc than control fibroblasts. Moreover, increased αSMA expression, cell migration, CTGF, and collagen release were observed in lipopolysaccharides-primed SSc fibroblasts after BzATP stimulation. While P2X7-induced cytokine changes did not affect collagen production, it was completely abrogated by inhibition of the ERK pathway.

**Conclusion:** In SSc fibroblasts, P2X7R is overexpressed and its stimulation induces Ca^2+^-signaling activation and a fibrogenic phenotype characterized by increased migration and collagen production. These data point to the P2X7R as a potential, novel therapeutic target for controlling exaggerated collagen deposition and tissue fibrosis in patients with SSc.

## Introduction

Systemic sclerosis (SSc; scleroderma) is a connective tissue disease (CTD) presenting with three major aspects: small vessel disease, autoantibody production, and fibroblasts dysfunction leading to exaggerated deposition of collagen and extracellular matrix (ECM), tissue fibrosis and organ dysfunction (Allanore et al., 2015). Clinical aspects and prognosis of SSc may diverge, most patients presenting skin thickening and changeable involvement of internal organs including lungs, heart, gastrointestinal tract, and kidneys (Allanore et al., 2015). Although, relatively rare (50–300 case per million), the disease has a relevant social impact since it typically affects young women leading to significant disability and mortality (Allanore et al., 2015). At the moment, no effective anti-fibrogenetic therapy is available for the disease (Allanore et al., 2015; Pattanaik et al., 2015).

Increasing evidence indicates that the purinergic system may play a key role in the fibrotic process, by regulating collagen and ECM production from the fibroblast. Although most studies focused on P1 adenosine receptors (Chan et al., 2006; Lazzerini et al., 2012; Perez-Aso et al., 2012, 2014), recent data suggest that also P2 purinergic receptors, particularly P2X7, may be actively involved in tissue fibrosis. The P2X7 receptor (P2X7R) is a nucleotide-gated ion channel chiefly involved in the inflammatory response triggered by the release of ATP from damaged cells. It is largely expressed in monocytes where it enhances the synthesis and/or promotes the release of lipopolysaccharide (LPS)-induced pro-inflammatory cytokines, particularly interleukin-1β (IL-1β) and interleukin-18, by activating the K+-dependent NALP3 inflammasome/caspase-1 pathway (Di Virgilio, 2007; Castrichini et al., 2014; Gicquel et al., 2015), but also possibly interleukin-6 (IL-6), via the Ca^2+^-dependent MAP-Kinase/early growth response-1 gene systems, egr-1 (Solini et al., 1999; Caporali et al., 2008; Friedle et al., 2011). Accumulating data demonstrated that the P2X7R is also expressed in fibroblastic cells (Solini et al., 1999; Caporali et al., 2008; Friedle et al., 2011), promoting lung, kidney, pancreas, and cardiac fibrosis in animal models (Gentile et al., 2015).

Starting from such premises, and taking into consideration that no information exists on a possible role of P2X7R in modulating fibrosis in scleroderma, we here hypothesized that P2X7R activation may stimulate the collagen biosynthetic process in SSc fibroblasts, thus representing a potential pharmacological target for the treatment of the disease. This study is aimed at determining expression and function of the P2X7R by evaluating calcium flux, α-SMA expression, cell migration, and collagen production in human skin fibroblast from SSc patients as compared to healthy controls.

## Materials and methods

### Skin biopsies

Dermal fibroblasts were prepared from 5-mm skin biopsies from the forearm of 9 SSc patients and 8 healthy volunteers matched for age and sex. The research received the approval by the Local Ethical Committee, and patients gave their informed consent in accordance with the Declaration of Helsinki. Demography of the subjects involved in the study is depicted in the Supplementary Table.

### Dermal fibroblasts isolation and culture

Skin specimens were digested using 1 mg/ml clostridial collagenase (Sigma-Aldrich, Milan, Italy) in phosphate buffered saline (PBS). Cell suspensions were plated out in 10 ml of Dulbecco's Modified Eagle Medium (DMEM) supplemented with L-glutamine (2 mM), fetal bovine serum (FBS; 10%), penicillin (200 U/ml) and streptomycin (200 μg/ml) in 100-mm culture dishes and incubated in a humidified atmosphere containing 5% CO_2_. The experiments were conducted at the third passage in order to avoid changes in the original phenotype. Except where indicated otherwise, all the reagents cited above were from Euroclone (Pero, Italy).

### Flow cytometry analysis of P2X7R expression

Fibroblasts (1 × 10^5^) were incubated with a specific polyclonal anti-P2X7R (extracellular) antibody fluorescein isothiocyanate (FITC) (Sigma-Aldrich, Milan, Italy) (Xie et al., 2014) for 30 min at 4°C. A rabbit IgG-FITC isotype control antibody was chosen to differentiate non-specific background fluorescence from specific antibody signal. Cells were analyzed with the Dako Galaxy Flow Cytometry System and the FlowMax software. The analysis was done on well-shaped living cells by setting FSC X SSC live gates that exclude cellular debris. After setting of appropriate gates 20,000 events were acquired for each sample. The expression of surface-labeled P2X7Rs was evaluated measuring both the percentage and the mean fluorescence intensity (MFI) of cells that were P2X7R positive using the FlowJo software, version 7.6.5. This analysis allows us to know the percentage of fibroblasts expressing P2X7R with respect to the total number of fibroblasts analyzed (% P2X7R+ cells) as well as the level of P2X7Rs expression per single cell, evaluated by its MFI.

### Analysis of P2X7R mRNA by qRT-PCR

Total RNA was extracted from fibroblasts (1 × 10^6^) using the RNeasy Mini kit (Qiagen S.p.A, Milano, Italy) following a standardized method (Del Ry et al., 2011). For cDNA synthesis, iScript cDNA Synthesis kit (Bio-Rad, Hercules, CA, USA) was used according to the manufacturer's instructions. Specific primers for P2X7R were synthesized by Qiagen (Hs_P2RX7_1_SG QuantiTect Primer Assay). Following recent guidelines (Vandesompele et al., 2002), three candidate reference genes [Eukaryotic translation elongation factor-1 alpha 1 (NM_001402), human ribosomal protein L13a (NM_012423) and ribosomal protein S4, X-Linked (NM_002046)], from among the most commonly cited in the literature, were selected and used to normalize mRNA expression data obtained by Real-Time PCR experiments. More details are provided in the Supplementary Material.

### P2X7R-induced calcium influx measurement

P2X7R is an ATP-gated cation channel. It acts as a bifunctional molecule whose brief activation causes the rapid (within milliseconds) and reversible opening of a channel selective for small cations, that induces the entry of Ca^2+^ and Na^+^ and the efflux of K^+^ while a sustained stimulation causes the opening of a larger pore. From a physiological point of view, it has been demonstrated that the channel opening mediates the effects of the P2X7R on cytokine production, in particular IL-1β maturation and release (triggered by the K^+^ efflux), and IL-6 expression (activated by the Ca^2+^ influx; North, 2002; Di Virgilio, 2007; Friedle et al., 2011). In the present study we studied the P2X7R functional activation in terms of Ca^2+^ influx, evaluated by the analysis of the cytosolic Ca^2+^ ([Ca^2+^]_*i*_) elevation. Indeed, since both Ca^2+^ influx and K^+^ efflux results from the opening of the same cationic channel, Ca^2+^ measurement was here employed as a marker more in general reflecting the extent of P2X7R (and related intracellular pathways) activation.

P2X7R activation was induced by using 2′-3′-O-(4-benzoylbenzoyl)ATP (BzATP) a selective P2X7R synthetic agonist that exhibits 10 fold greater potency respect to the physiological activator ATP (Jacobson, 2010). Technical details are provided as Supplementary Material.

In order to describe the features of Ca^2+^ influx, the following parameters were measured: (i) the Area Under the Curve (AUC) as an expression of the area included under the curve of the [Ca^2+^]i over the time for 5 min following the agonist addition [AUC (5′)] and (ii) the percentage (%) of responsive cells.

### Culture stimulation

Dermal fibroblasts were plated out in 500 μl of starvation medium (DMEM plus 0.5 % FBS). After 24 h in starvation, cells were primed with lipopolysaccharide (LPS, 1 μg/ml) from *E. coli* 026:B6 or vehicle for 24 h, and then stimulated with the P2X7R agonist BzATP (0.1 mM) for 30 min. Cells were also stimulated with LPS alone.

In order to confirm the specific involvement of the P2X7R in the observed effects, experiments with two different synthetic P2X7R antagonists were performed, by incubating for 2 h the LPS-primed cells with oxidized ATP (oATP, 200 μM) or A438079 (10–50 μM) before BzATP treatment.

The putative effects of IL-6 released by SSc fibroblasts upon P2X7R activation was investigated adding the recombinant human soluble IL6 receptor (IL6α, 10 ng/ml) to the BzATP+LPS-stimulated cells for 24 h.

Finally, the involvement of ERK-1/2 pathway in P2X7R activation-induced collagen production was evaluated by pre-treatement of the fibroblasts with the selective ERK-1/2 inhibitor FR-180204 (50 μM) for 30 min before BzATP+LPS stimulation.

LPS, BzATP, and oATP were purchased from Sigma-Aldrich (Milan, Italy). FR-180204, A438079 and soluble IL-6Rα from Tocris (Bristol, UK). Reagents were dissolved in dimethyl sulfoxide (DMSO, Sigma-Aldrich, Milan, Italy) or in distilled water. Accordingly, fibroblast cultures treated with the appropriate vehicle were used as basal samples.

### Collagen analysis

Collagen concentration was determined in the supernatant of the cells collected at the end of each treatment and stored at −20°C until used. By means of the EIA kit (Takara Bio Inc., Otsu, Japan) we evaluated the procollagen type I carboxy-terminal peptide (PIP) released in the culture supernatants as an expression of the collagen synthesis. PIP levels were measured as ng/ml. Technical details are reported as Supplementary Material.

### Immunofluorescence experiments

Fibroblasts were seeded on 13 mm glass coverslip and stimulated or not with LPS and BzATP in the presence or in the absence of A438079 (50 μM), as described in culture stimulation section. The cells were then fixed with paraformaldehyde 4% for 15′ at RT and permeabilized with Triton X-100 0.1% for 10′ at RT. After blocking with 1%BSA, 10% goat serum, 0.1% Tween-20 and 2 mM EDTA for 1 h at RT, the cells were incubated with a mouse anti-α smooth muscle actin (αSMA) antibody (Sigma-Aldrich, Saint Louis, MO, USA) 1:200, overnight at 4°C and then reacted with a goat anti-mouse cy3 1:5000 (Jackson Laboratories, Bar Harbor, ME). Nuclei were visualized by 4′,6-diamidino-2-phenylindole (DAPI, Sigma-Aldrich). After mounting the cells were examined in a fluorescence microscope Nikon Eclipse Ti coupled to a DS-Q1Mc camera and a NIS element software (Nikon, Tokio, Japan).

### Scratch wound healing assay

Fibroblasts were plated onto 24-well plates allowed to grow to confluency in the same medium as for cell culture. Once confluent, the medium was switched to starvation medium (DMEM plus 0.2% FBS) for 24 h to minimize the influence of hormones and growth factors. After the starvation period, the cells were scratched with a 200 μl pipette tip, washed two times with PBS to remove cellular debris after that treatments were applied. Wells were imaged at 0 and 48 h at 4x magnification with phase contrast Zeiss Axiocam. Image J was used to measure the area of the scratch remaining at the different time points. The free hand tool on Image J was used to draw around the scratch and area in mm^2^ was measured after setting the field of view to 3,400 × 2,700 μm.

### Cytokine supernatant assay

IL-1β and IL-6 cytokines release was analyzed in the culture supernatants using commercially available Bio-Plex-Pro Human Cytokine assay (BioRad Laboratories, Hercules, CA) according to the manufacturer's protocol. Sample were acquired by a Bio-Plex 200 system and after setting up the calibration the cytokines concentration were performed using the Bio-Plex Manager software v5.0 (BioRad).

### Connective tissue growth factor (CTGF) supernatant assay

CTGF concentration was measured in duplicate in culture supernatants of dermal fibroblasts from healthy controls and SSc patients after different treatments, by using the Human CTGF ELISA Kit (Cloude-Clone Corp., TX, USA) according to the manufacturer's instructions.

### Statistical analysis

All experiments were performed in duplicate or triplicate. Normal distribution of quantitative variables was preliminarily tested using the Kolmogorov–Smirnov test to select parametric (normal distribution) or non-parametric (non-normal distribution) inferential statistical methods. Accordingly, the following parametric or non-parametric statistical analyses were performed, respectively: the two-tailed unpaired *t*-test or the two-tailed Mann–Whitney test to evaluate differences in continuous variables between two groups. The repeated measures analysis of variance (RM-ANOVA) and the two-tailed Tukey–Kramer *post-hoc* multiple comparisons test, or the Friedman test (non-parametric RM-ANOVA), or Kruskal–Wallis test (one-way non-parametric ANOVA) and the two-tailed Dunn's *post-hoc* multiple comparison test to evaluate differences in continuous variables among more than two groups. Values of *p* < 0.05 were considered significant. All statistical analyses were done using GraphPad-InStat package (GraphPad, La Jolla, CA, USA).

## Results

### P2X7R surface expression is increased in SSc dermal fibroblasts

**Figure 1:**
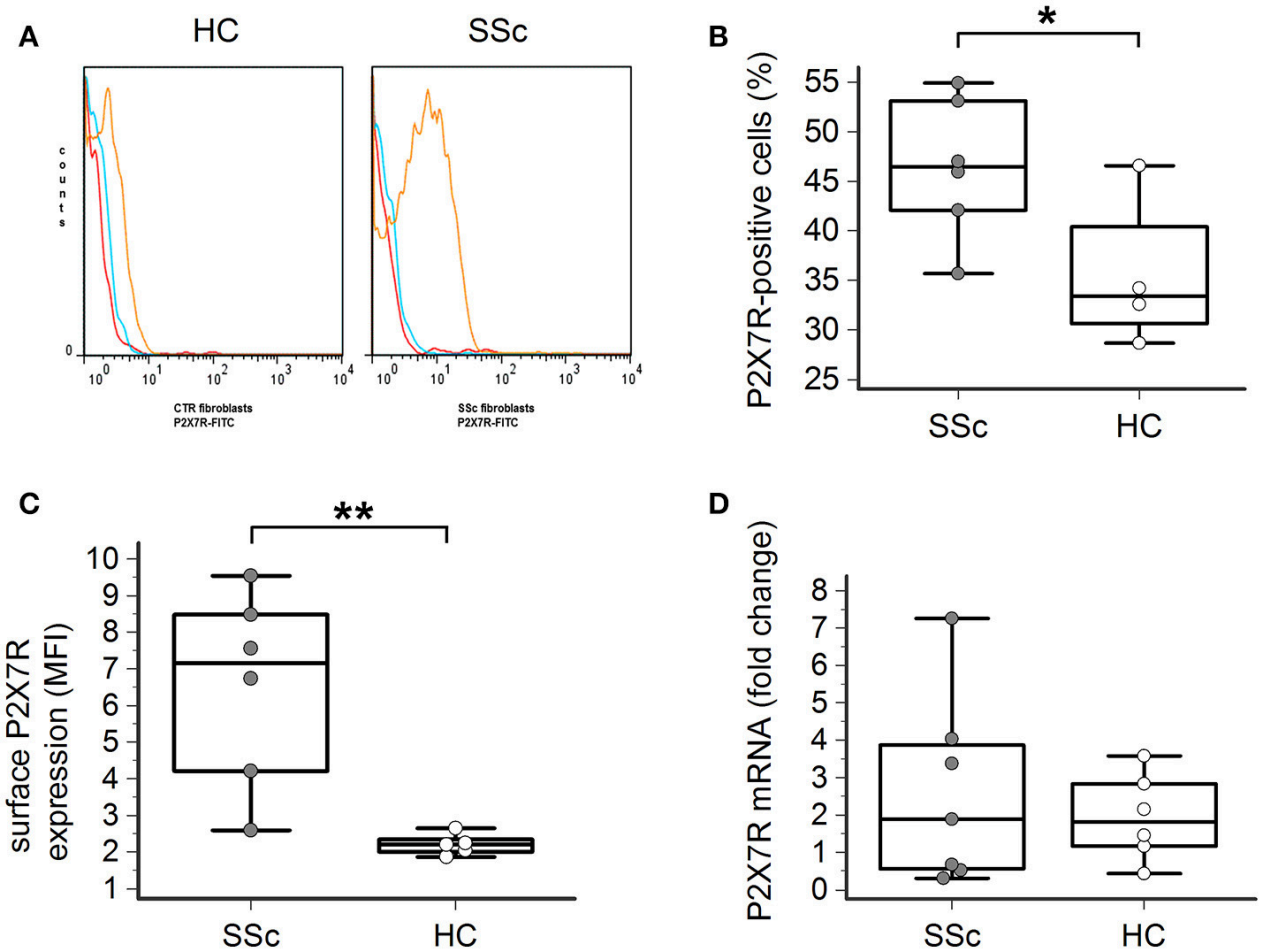
P2X7R surface expression is increased in SSc dermal fibroblasts. **(A–C)** P2X7R surface expression in dermal fibroblasts from SSc patients and HC, as determined by flow cytometry: **(A)** original flow cytometry data representative of an experiment; **(B)** percentage of P2X7R positive cells, and **(C)** MFI values. SSc patients, *n* = 6; HC, *n* = 5. ^*^p < 0.05, two-tailed unpaired *t*-test; ^**^p < 0.01, two-tailed Mann–Whitney test. **(D)** P2X7R mRNA expression in dermal fibroblasts from SSc patients and HC, as determined by quantitative RT-PCR analysis. SSc patients, *n* = 7; HC, *n* = 6. *p* = not significant, two-tailed unpaired *t-*test. SSc, Systemic sclerosis; HC, healthy controls; P2X7R, P2X7 receptor; MFI, mean fluorescence intensity; RT-PCR, reverse transcription-polymerase chain reaction.

Flow cytometry analysis showed that the P2X7R was expressed on the surface of both healthy and SSc dermal fibroblasts (Figures 1A–C). However, both the percentage of P2X7-positive cells (46 ± 7 vs. 35 ± 6%, *p* = 0.023; Figure 1B) and, particularly, the total amount of the P2X7R expression was significantly higher in SSc than healthy fibroblasts, as demonstrated by the ~3-times higher values of mean fluorescence intensity (MFI; 6.5 ± 2.6 vs. 2.2 ± 0.3, *p* = 0.0087; Figure 1C).

Such a phenomenon was not associated with an increased P2X7R gene expression by the cells, as real time-PCR analysis indicated that the levels of P2X7R mRNA were not significantly different in SSc patients when compared to healthy controls (2.6 ± 2.5 vs. 1.9 ± 1.1 fold-increase, *p* = 0.58; Figure 1D).

### P2X7R-mediated calcium influx is enhanced in dermal fibroblasts from SSc patients

**Figure 2:**
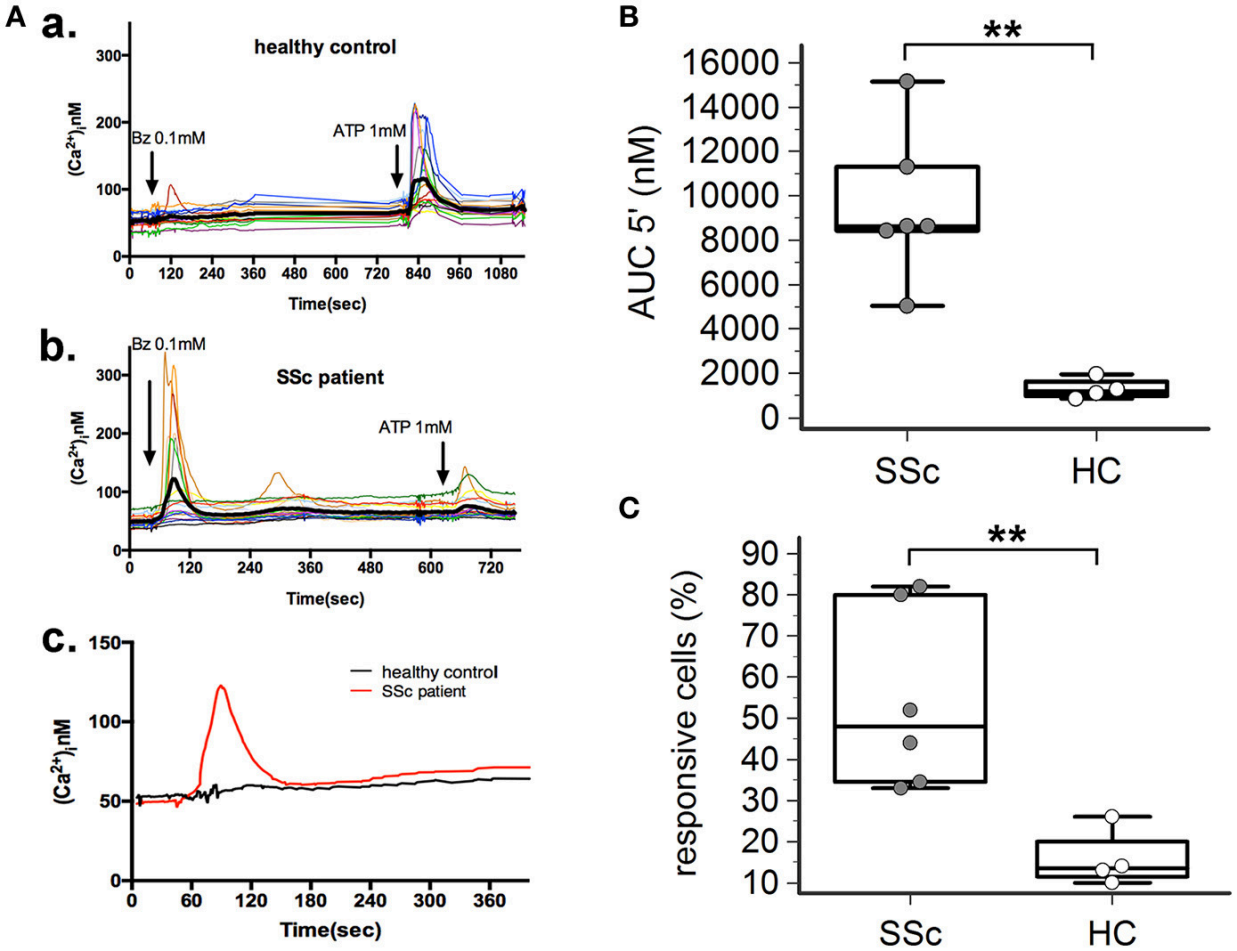
P2X7R-mediated calcium influx is enhanced in dermal fibroblasts from SSc patients. BzATP-induced calcium entry in dermal fibroblasts from SSc patients and HC, as determined by single-cell fluorescence microscopy. **(A)** Representative plot of an experiment in one SSc patient **(a,c)** and one HC **(b,c)**. Each colored trace in **(a,b)** represents the response of individual cells while the thick black line in **(a,b)**, and the red and black lines in **(c)** represent the mean response of all the cells from the same culture dish. The arrow indicates the time when the agonist was added. **(B)** P2X7-induced Ca2+ uptake as assessed by the AUC 5′, in dermal fibroblasts from SSc patients and HC. SSc patients, *n* = 6; HC, *n* = 4. ^**^p < 0.01, two-tailed Mann–Whitney test. **(C)** Percentage of BzATP-responsive cells in SSc patients and HC. SSc patients, *n* = 6; HC, *n* = 4. ^**^p < 0.01, two-tailed Mann–Whitney test. SSc, Systemic sclerosis; HC, healthy controls; P2X7R, P2X7 receptor; Bz, 2′-3′-O-(4-benzoylbenzoyl)ATP; Ca2+, calcium; AUC 5′, area under the curve represents the area enclosed between the curve of the [Ca^2+^]_ì_vs. the time for 5 min after the addition of the stimulus.

**Figure 3:**
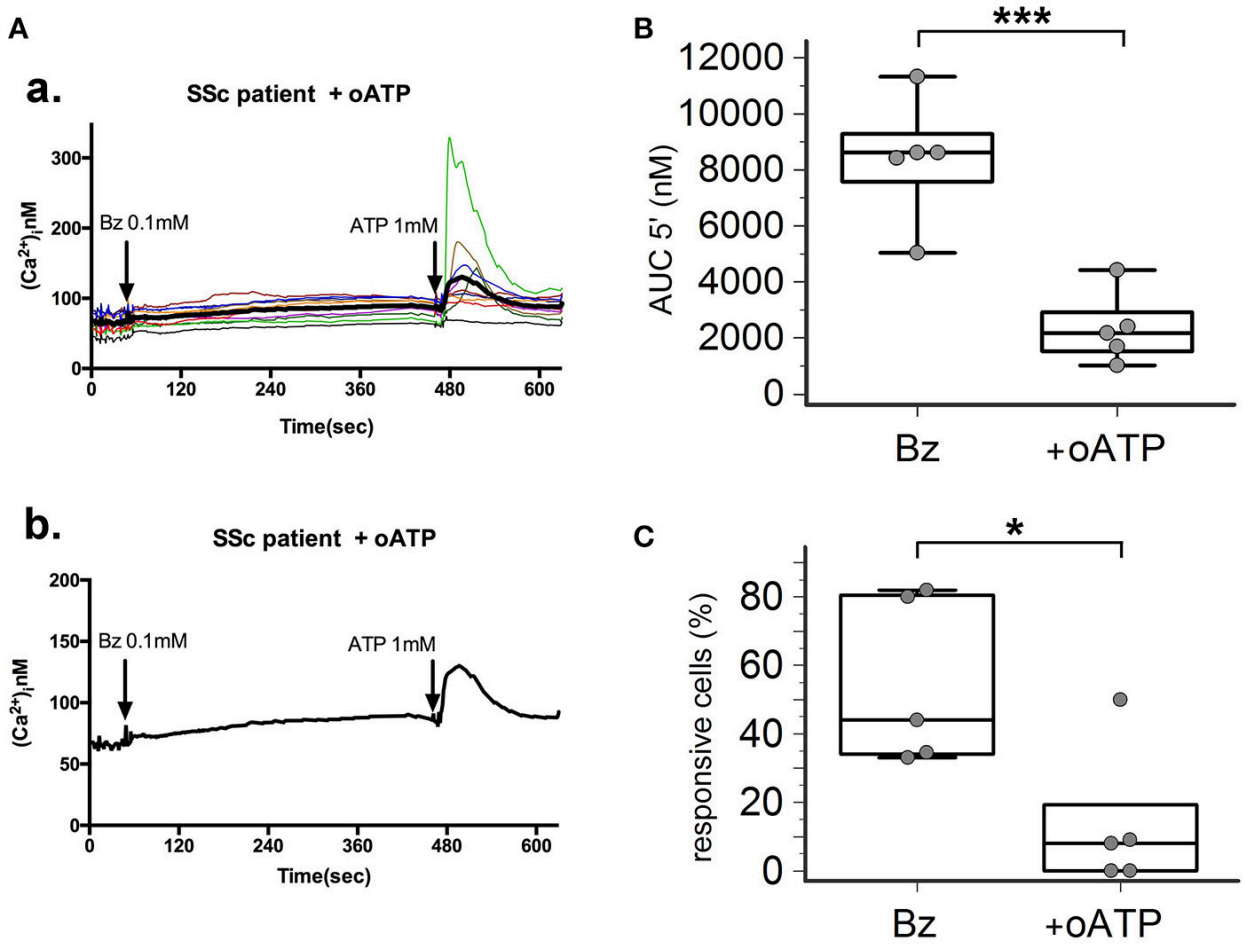
P2X7R antagonism inhibits calcium influx in SSc dermal fibroblasts. Effect of the P2X7R antagonist oxATP (200 μM) on BzATP-induced calcium entry in dermal fibroblasts from SSc patients, as determined by single-cell fluorescence microscopy. **(A)** Representative plot of an experiment in one SSc patient **(a,b)**. Each colored trace in **(a)** represents the response of individual cells while the thick black line in **(a,b)** represent the mean response of all the cells from the same culture dish. The arrow indicates the time when the agonist was added. **(B)** P2X7-induced Ca2+ influx in dermal fibroblasts from SSc patients (*n* = 5) pre-incubated with oATP, as assessed by the AUC 5′. ^***^p < 0.01, two-tailed paired *t-*test. **(C)** Percentage of BzATP-responsive cells in SSc patients (*n* = 5) pre-incubated with oATP. ^*^p < 0.05, two-tailed paired *t*-test. SSc, Systemic sclerosis; P2X7R, P2X7 receptor; Bz, 2′-3′-O-(4-benzoylbenzoyl)ATP; oATP, oxidized ATP; Ca2+, calcium; AUC 5′, area under the curve represents the area enclosed between the curve of the [Ca^2+^]_ì_vs. the time for 5 min after the addition of the stimulus.

P2X7R stimulation with the receptor agonist BzATP resulted in calcium influx in normal and SSc dermal fibroblasts in the presence of extracellular Ca^2+^ (Figure 2A). However, significant differences between the two cell populations were found, indicative of an increased P2X7-dependent Ca^2+^ permeability in SSc fibroblasts. In fact, both the number of responding cells (54 ± 22 vs. 16 ± 7%, *p* = 0.0095; Figure 2C), and the extent of Ca^2+^ uptake as assessed by the AUC (9,527 ± 3,396 vs. 1,293 ± 477 nM, *p* = 0.0093; Figure 2B) were significantly higher when SSc cells were compared to healthy subject fibroblasts. Notably, pre-incubation of SSc fibroblasts with the P2X7R antagonist oATP significantly reduced BzATP-induced calcium influx, to values substantially comparable to controls (number of responding cells: 13±21%, *p* = 0.015; AUC: 2,342 ± 1,280 nM, *p* = 0.0003; Figure 3).

### P2X7R activation stimulates collagen production in SSc dermal fibroblasts

**Figure 4:**
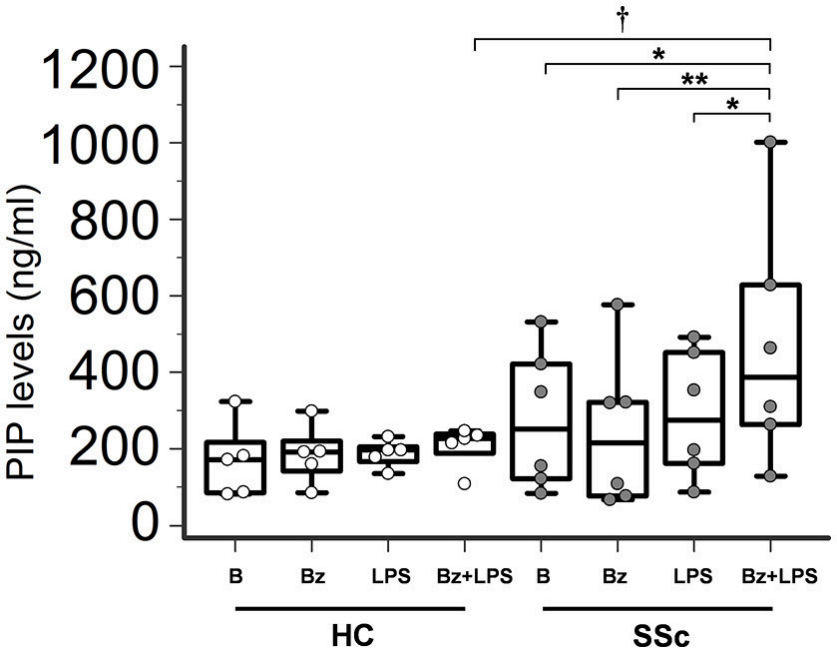
P2X7R activation stimulates collagen production in SSc dermal fibroblasts. Spontaneous, Bz-, LPS-, and Bz+LPS-induced PIP release in the culture medium of dermal fibroblasts from SSc patients and HC. Cells starved for 24 h were primed with LPS (1 μg/ml) or vehicle for further 24 h, and then stimulated with Bz (0.1 mM) for 30 min; cells were also stimulated with LPS alone (24 h). SSc patients, *n* = 6; HC, *n* = 5. Comparisons within SSc patients: RM-ANOVA, *p* = 0.0064; *post-hoc* two-tailed Tukey–Kramer test, ^*^p < 0.05, ^**^p < 0.01. Comparisons within HC: Friedman test (non-parametric RM-ANOVA), *p* = not significant. Comparison between SSc patients and HC: †*p* < 0.05, two-tailed Mann–Whitney test. SSc, Systemic sclerosis; HC, healthy controls; P2X7R, P2X7 receptor; B, baseline; Bz, 2′-3′-O-(4-benzoylbenzoyl)ATP; LPS, lipopolysaccharide; PIP, procollagen type I carboxy-terminal peptide; RM-ANOVA, repeated measures analysis of variance.

**Figure 5:**
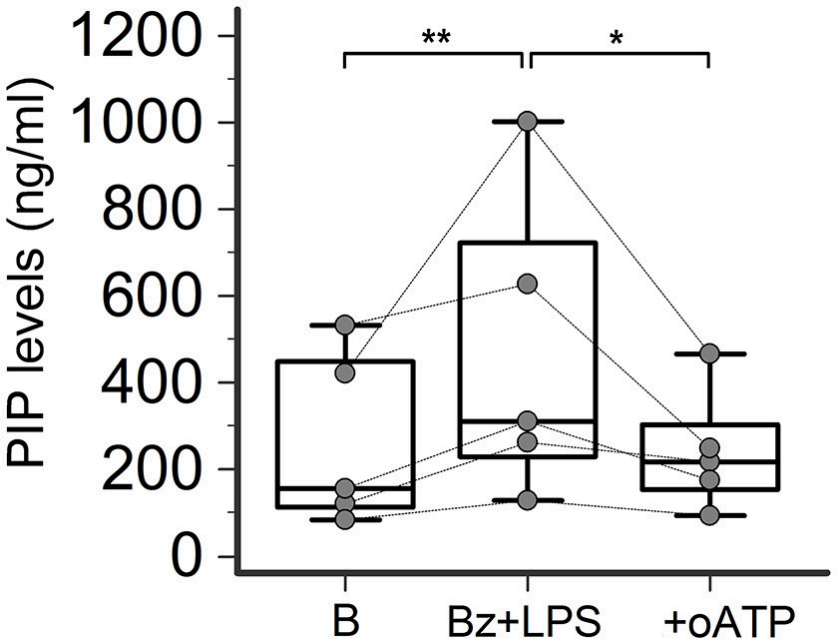
P2X7R antagonism inhibits collagen production in SSc dermal fibroblasts. Effect of the P2X7R antagonist oATP (200 μM) on Bz+LPS-induced PIP release in the culture medium of dermal fibroblasts from SSc patients, as determined by EIA assay. SSc patients, *n* = 5. RM-ANOVA, *p* = 0.0052; *post-hoc* two-tailed Tukey–Kramer test, ^*^p < 0.05, ^**^p < 0.01. SSc, Systemic sclerosis; HC, healthy controls; P2X7R, P2X7 receptor; B, baseline; Bz, 2′-3′-O-(4-benzoylbenzoyl)ATP; LPS, lipopolysaccharide; oATP, oxidized ATP; PIP, procollagen type I carboxy-terminal peptide; RM-ANOVA, repeated measures analysis of variance.

In LPS-primed fibroblasts from SSc patients, BzATP-dependent P2X7R activation resulted in a clear-cut stimulation of collagen synthesis. As shown in the Figure 4, while cell stimulation with LPS or BzATP alone produced no changes in PIP release, LPS plus BzATP significantly increased supernatant PIP levels in SSc dermal fibroblasts (466.0 ± 313.9 vs. 277.0 ± 183.1 ng/ml in untreated cells, +76.7%; *p* < 0.05). This effect was not due to an increase in cells number after stimulation (62 ± 17.6 vs. 62.89 ± 22.8 cells/microscopic field analyzed in parallel experiments). Such a stimulating effect was completely removed when cultures were co-incubated with the P2X7R antagonist, oATP (Figure 5). On the contrary, none of the treatments induced appreciable effects on collagen synthesis in healthy subject cells.

### P2X7R stimulation increases αSMA bundles expression in SSc dermal fibroblasts

Differentiation of fibroblast into myofibroblast can be marked by several morphological and functional changes, including cell dimension increase, collagen production and expression of contractile proteins such as αSMA. In particular, a well-recognized characteristic of the fully differentiated myofibroblast is the accumulation of cytoplasmatic microfilaments constituted by αSMA bundles (stress fibers) with high contractile activity (Talele et al., 2015; Garrett et al., 2017).

**Figure 6:**
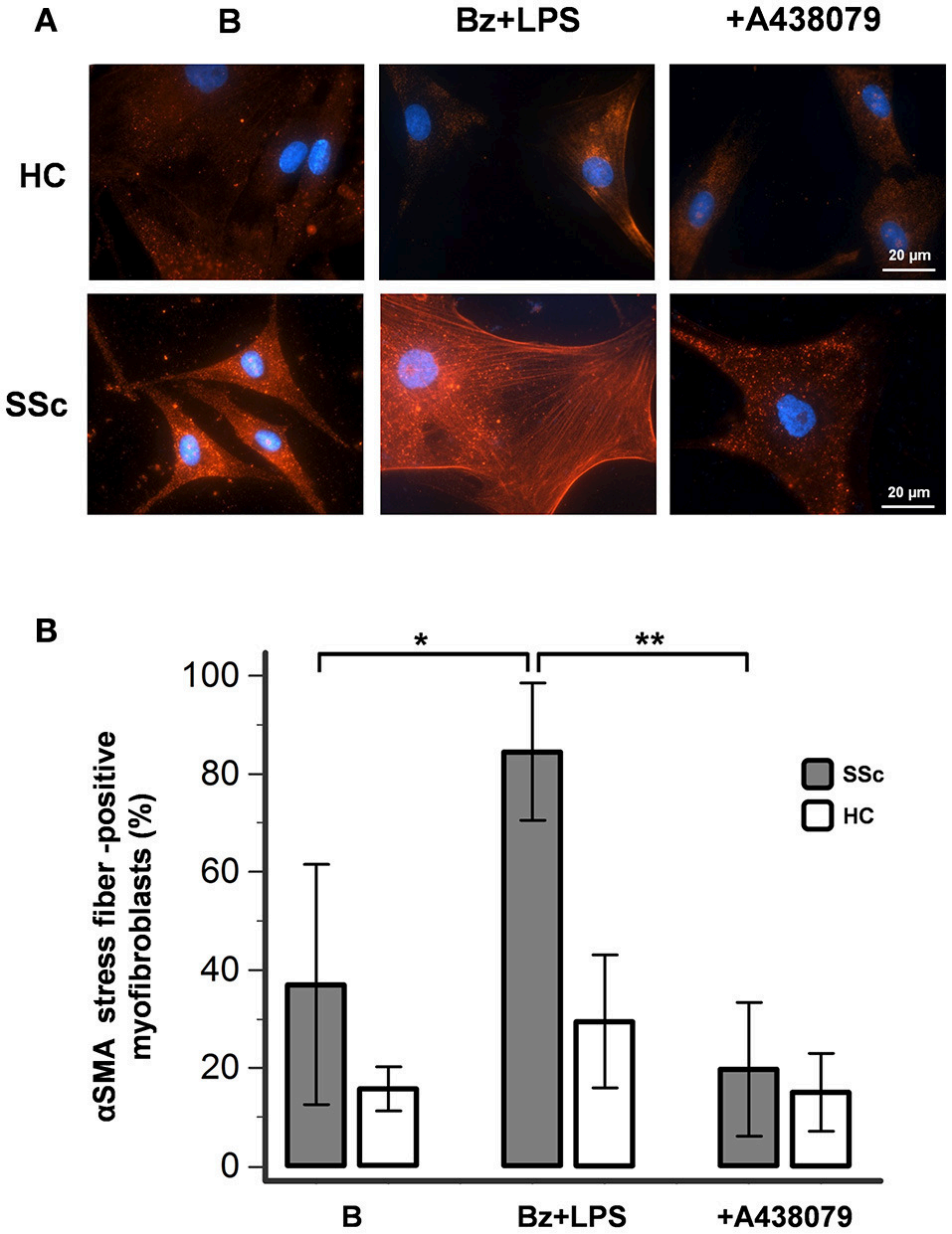
P2X7R activation promotes myofibroblast differentiation of SSc dermal fibroblasts. Effect of Bz+LPS stimulation on the differentiation of dermal fibroblasts to myofibroblasts, identified as large size and αSMA bundle (stress fiber)-positive cells by immunofluorescence. Fibroblasts from SSc patients and HC were starved for 24 h and then primed with LPS (1 μg/ml) or vehicle for further 24 h, followed by stimulation with Bz (0.1 mM) for 30 min, alone or after 2 h pre-incubation with the P2X7R antagonist A438079 (50 μM). **(A)** Representative figure of an experiment performed in SSc and HC fibroblasts, respectively: in red the immunofluorescence for α-SMA, while nuclei were stained in blue with DAPI. Images were acquired with a 60X/1.49 Apo objective. **(B)** Effect of Bz+LPS and Bz+LPS+AR438079 on myofibroblast differentiation, as determined by the percentage of αSMA stress fiber-positive cells. Histograms represent results (mean ± standard deviation) of experiments performed by using fibroblasts from SSc patients (*n* = 4) and HC (*n* = 3). RM-ANOVA, *p* = 0.012; *post-hoc* two-tailed Tukey–Kramer test, ^*^p < 0.05, ^**^p < 0.01. P2X7R, P2X7 receptor; SSc, Systemic sclerosis; B, baseline; Bz, 2′-3′-O-(4-benzoylbenzoyl)ATP; LPS, lipopolysaccharide; HC, healthy controls; DAPI, 4′,6-diamidino-2-phenylindole; ANOVA, analysis of variance.

In basal conditions, healthy fibroblasts had a very weak reaction for αSMA when compared to SSc dermal fibroblasts (Figure 6). However, despite showing a high fluorescence intensity, SSc cells displayed a heterogeneous αSMA staining represented by a punctate and bundled pattern, with a prevalence of cells with punctate distribution (63 ± 24% of cells counted in at least five random fields of 3 separate coverslips for each of 4 different patients). P2X7R stimulation with LPS + BzATP markedly increased the percentage of large-size fibroblasts expressing well-developed αSMA microfilament bundles (from 37 ± 24 to 84 ± 14%; *p* < 0.05). Conversely, in the presence of the P2X7R inhibitor A438079, the percentage of αSMA stress fiber-positive cells significantly fell below the basal levels (19 ± 14%; Figure 6), thus suggesting a constitutive activity of the receptor in the SSc fibroblast. Culture stimulation with LPS + BzATP induced a slight increase of αSMA stress fiber-positive cells also in healthy fibroblasts (from 16 ± 4 to 30 ± 14%), an effect which was completely prevented by A438079 pre-incubation (15 ± 8%; Figure 6).

### P2X7R stimulation increases SSc dermal fibroblasts migration

**Figure 7:**
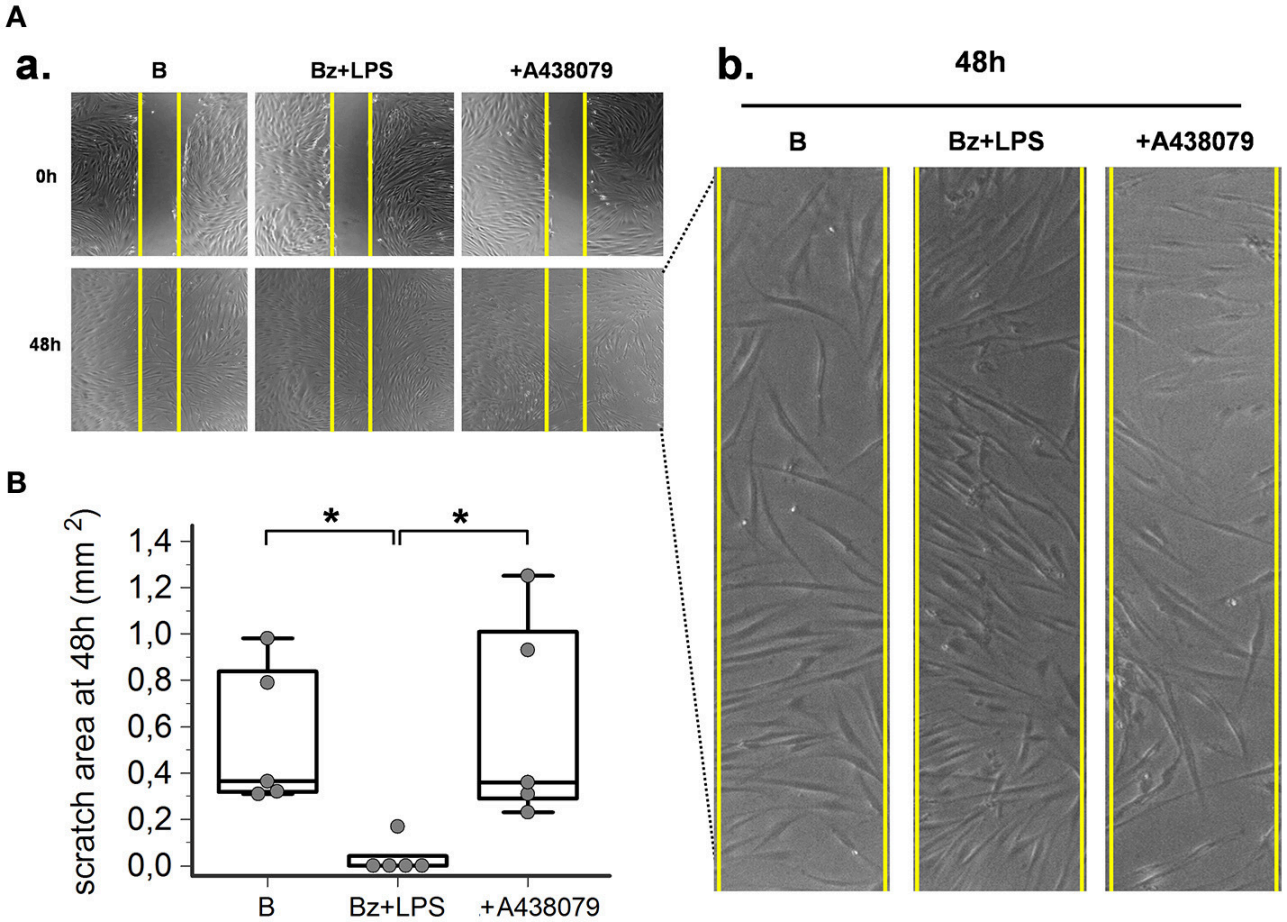
P2X7R stimulation increases SSc fibroblasts migration. P2X7R-dependent migration of SSc dermal fibroblasts, as determined by *in vitro* scratch-wound healing assays. **(A) (a)** Representative figure of an experiment. Cells are stimulated with Bz+LPS alone or in association with the P2X7R antagonist A438079 (50 μM). Photos were taken immediately after wounding (0 h) and 48 h later. Yellow vertical lines show the scratch area, as initially measured at 0 h. **(b)** Magnification of the scratch area at 48 h. **(B)** Effect of Bz+LPS and Bz+LPS+AR438079 on SSc dermal fibroblast migration, as determined by the scratch area at 48 h. Plots represent 5 different experiments performed by using fibroblasts from 3 different SSc patients. Kruskal–Wallis test (non-parametric ANOVA), *p* = 0.0083; *post-hoc* two-tailed Dunn multiple comparison test, ^*^p < 0.05. SSc, Systemic sclerosis; P2X7R, P2X7 receptor; B, baseline; Bz, 2′-3′-O-(4-benzoylbenzoyl)ATP; LPS, lipopolysaccharide; ANOVA, analysis of variance.

As predicted by the enhanced expression of contractile proteins, wound healing experiments performed *in vitro* by using a scratch assay demonstrated that P2X7R also increased SSc fibroblast migration. After LPS priming, P2X7R stimulation with BzATP promoted wound healing as demonstrated by the increased SSc cell migration to closing the wound scratch (Figures 7A,B). This view was further confirmed by the evidence that culture co-incubation with the P2X7R-inhibitor A438079 completely abrogated the effect (Figures 6A,B).

### P2X7R activation induces IL-6, but not IL-1β, release from SSc dermal fibroblasts

**Figure 8:**
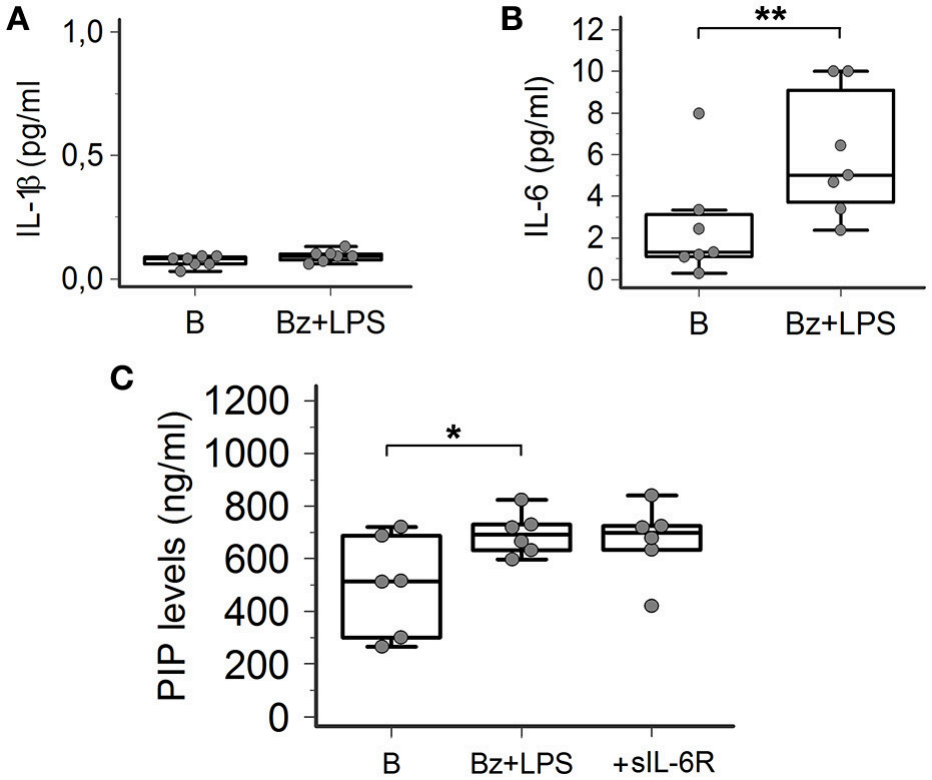
P2X7R activation induces IL-6 release from SSc dermal fibroblasts, but the cytokine does not contribute to collagen production. **(A)** Effect of Bz+LPS stimulation on the IL-1β release in the culture medium of dermal fibroblasts from SSc patients; *n* = 7. *p* = not significant, two-tailed paired *t-*test. **(B)** Effect of Bz+LPS stimulation on the IL-6 release in the culture medium of dermal fibroblasts from SSc patients; *n* = 7. ^**^p < 0.01, two-tailed paired *t-*test. **(C)** Effect of the addition of the sIL-6R on Bz+LPS-induced PIP release in the culture medium of dermal fibroblasts from SSc patients, as determined by EIA assay; *n* = 6. RM-ANOVA, *p* = 0.023; *post-hoc* two-tailed Tukey–Kramer test, ^*^p < 0.05. SSc, Systemic sclerosis; P2X7R, P2X7 receptor; B, baseline; Bz, 2′-3′-O-(4-benzoylbenzoyl)ATP; LPS, lipopolysaccharide; IL-1β, interleukin-1β; IL-6, interleukin-6; sIL-6R, soluble interleukin-6 receptor; PIP, procollagen type I carboxy-terminal peptide; RM-ANOVA, repeated measures analysis of variance.

In order to provide information about the possible intracellular mechanisms involved in P2X7R-dependent collagen production in LPS-primed SSc dermal fibroblasts, we evaluated whether the LPS plus BzATP co-incubation was associated with the production of specific cytokines, particularly IL-1β and IL-6, as a possible result of the P2X7R-induced activation of the K^+^-dependent NALP3 inflammasome/caspase-1 or the Ca^2+^-dependent MAP-Kinase/egr-1 systems, respectively. As shown in Figures 8A,B, while no changes in IL-1β were observed (0.07 ± 0.02 vs. 0.09 ± 0.02 pg/ml; *p* = 0.41), co-treatment of SSc cells with LPS + BzATP induced a significant 2.4-fold increase in IL-6 supernatant levels (2.5 ± 2.6 vs. 6.0 ± 3.0 pg/ml; *p* = 0.0032).

### IL-6 does not contribute to P2X7R-dependent collagen production from SSc dermal fibroblasts

Since human dermal fibroblasts (thus SSc fibroblasts) do not express on their surface the IL-6 receptor (transmembrane IL-6R, mIL-6R) required for activating classic signaling (Lazzerini et al., 2016), the possibility can be ruled out that the P2X7-dependent stimulating effect on collagen production observed *in-vitro* is the result of the above reported IL-6 increase, via an autocrine mechanism. Nevertheless, we evaluated the potential additive role of the P2X7R-dependent IL-6 increase in enhancing collagen production from SSc fibroblast via the soluble IL-6R (sIL-6R)-dependent transsignaling pathway, potentially relevant in SSc patients *in-vivo*. In fact, differently from *in vitro* conditions, the sIL-6R is freely available *in vivo* in human plasma where it is released by hepatocytes, leukocytes and megakaryocytes (Lazzerini et al., 2016). On these premises, LPS-primed SSc fibroblasts were stimulated with BzATP in the presence of sIL-6R, but no any further increase in collagen supernatant levels was observed (Figure 8C).

### P2X7R activation induces CTGF release from dermal fibroblasts

**Figure 9:**
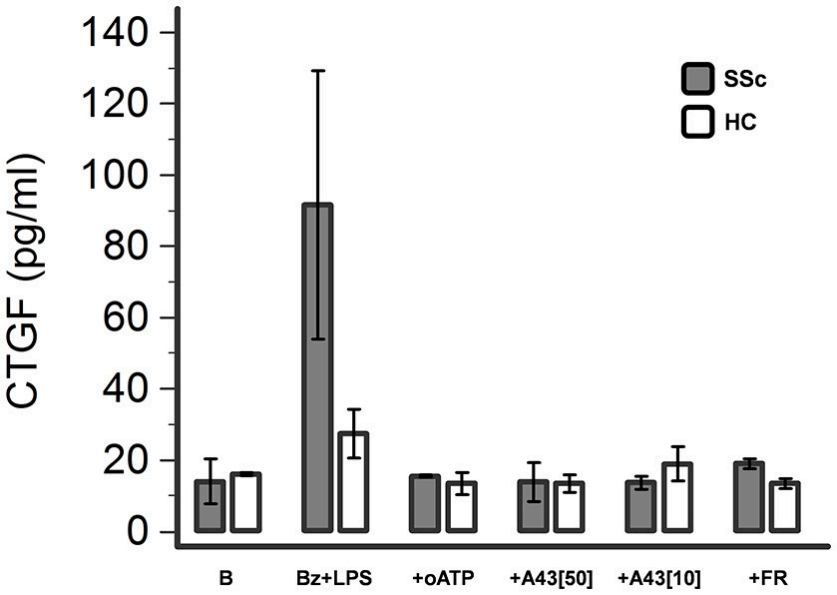
P2X7R stimulation induces CTGF release from dermal fibroblasts, which is completely abrogated by P2X7R antagonism or ERK-1/2 inhibition. Effect of Bz+LPS stimulation on CTGF release in the culture medium of dermal fibroblasts from SSc patients (*n* = 2) and healthy controls (*n* = 2), as determined by ELISA assay, and its modulation by P2X7R antagonists (oATP 200 μM; A438079 10–50 μM) or an ERK-1/2 inhibitor (FR-180204 50 μM). CTGF, connective tissue growth factor; SSc, Systemic sclerosis; P2X7R, P2X7 receptor; B, baseline; Bz, 2′-3′-O-(4-benzoylbenzoyl)ATP; LPS, lipopolysaccharide; oATP, oxidized ATP; A43[50], A438079 50 μM; A43[10], A438079 10 μM; FR, FR-180204.

The possible pathway involved in mediating P2X7R-induced SSc fibroblast activation was then further investigated by evaluating the effect of LPS + BzATP stimulation on the release of CTGF in the culture supernatant. Under these conditions, CTGF levels showed a clear-cut increase (~6-fold, from 13.9 ± 6.3 to 91.6 ± 36.7 pg/ml) which was completely abrogated by culture pre-incubation with the P2X7 antagonists oATP (15.4 ± 0.4 pg/ml) or A438079 (50 μM: 13.9 ± 5.4 pg/ml; 10 μM:13.6 ± 1.8 pg/ml; Figure 9). CTGF release was also affected in healthy fibroblasts, although to a lesser extent: culture stimulation with LPS + BzATP induced a ~2-fold CTGF increase (from 16.0 ± 0.4 to 27.4 ± 6.9 pg/ml), an effect which was completely prevented by oATP (13.4 ± 3.1 pg/ml) or A438079 (50 μM: 13.5 ± 2.5 pg/ml; 10 μM: 18.9 ± 4.8 pg/ml) pre-incubation (Figure 9).

### ERK-1/2 inhibition completely prevents P2X7R-induced collagen production in SSc dermal fibroblasts

**Figure 10:**
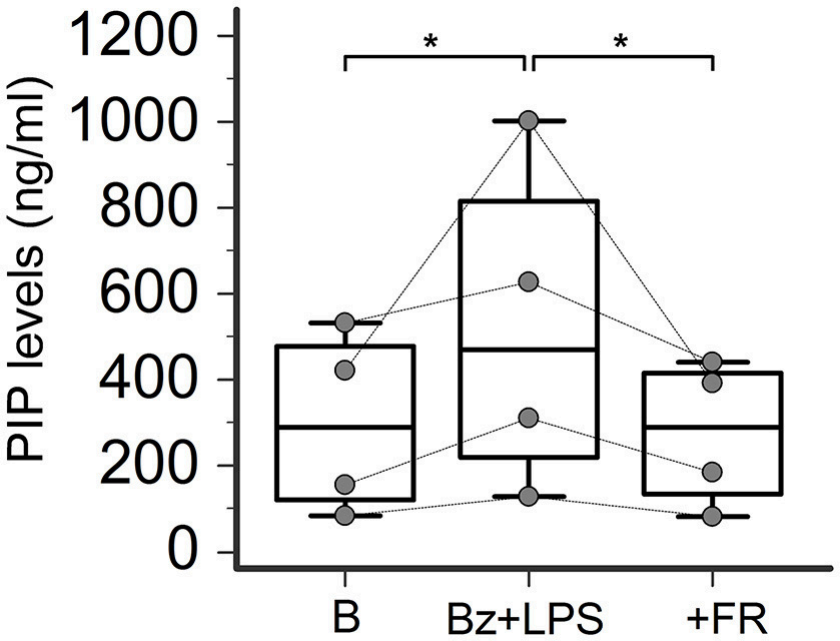
ERK-1/2 inhibition completely prevents P2X7R-induced collagen production in SSc dermal fibroblasts. Effect of the ERK-1/2 inhibitor FR-180204 (50 μM) on Bz+LPS-induced PIP release in the culture medium of dermal fibroblasts from SSc patients, as determined by EIA assay. SSc patients, *n* = 4. RM-ANOVA, *p* = 0.013; *post-hoc* two-tailed Tukey–Kramer test, ^*^p < 0.05. SSc, Systemic sclerosis; P2X7R, P2X7 receptor; ERK-1/2, extracellular signal-regulated kinases-1/2; B, baseline; Bz, 2′-3′-O-(4-benzoylbenzoyl)ATP; LPS, lipopolysaccharide; FR, FR-180204; PIP, procollagen type I carboxy-terminal peptide; RM-ANOVA, repeated measures analysis of variance.

Increasing evidence indicates that LPS-besides constituting an essential stimulus for the activation of the NALP3 inflammasome via a Toll-like receptor-mediated mechanism- can also directly interact with the P2X7R by recognizing a sequence of the carboxy-end which contains a conserved LPS-binding domain (Denlinger et al., 2001). This interaction is required to induce LPS-dependent activation of the extracellular signal-regulated kinases (ERK1, ERK2; Denlinger et al., 2001), which in turn are involved in SSc fibroblast activation leading to collagen production (Bogatkevich et al., 2007; Chen et al., 2008). Thus, we evaluate the possible involvement of this pathway as the intracellular mechanism linking P2X7R and collagen production in LPS-primed SSc fibroblasts by pre-treating the cells with the ERK-1/2 inhibitor, FR-180204. Figure 10 shows that the addition of this molecule to the cultures completely abrogated the P2X7R-dependent stimulating effect on collagen production, as assessed by supernatant PIP levels.

Notably, also P2X7R-induced CTGF release was fully reversed when fibroblast cultures were pre-incubated with FR-180204 (Figure 9).

## Discussion

The main findings of the present study are the following: (i) when compared to healthy controls, dermal fibroblasts from SSc patients display a higher P2X7R surface expression with an enhanced function in terms of Ca^2+^ influx; (ii) in LPS-primed SSc fibroblasts, P2X7R stimulation results in profibrotic effects by promoting enhanced αSMA expression, cell migration, CTGF, and collagen production; (iii) intracellular mechanisms underlying P2X7R-induced fibrogenic effects seem to involve a cytokine-independent pathway likely due to ERK-1/2 signaling activation. Altogether, these data point to the P2X7R as a potential, novel therapeutic target for controlling exaggerated collagen deposition and tissue fibrosis in patients with SSc.

The P2X7 protein is a 595 amino acids sequence consisting of an intracellular N-terminus, two hydrophobic transmembrane domains, an extracellular loop, and an intracellular C-terminus (North, 2002). The main structurally distinctive feature of the P2X7R is a long C-terminal tail (244 amino acids) that is essential for pore formation, receptor trafficking and stabilization of the P2X7R in the membrane. Moreover, it harbors multiple potential protein and lipid interaction motifs (Vandesompele et al., 2002; Smart et al., 2003). Although the most recognized role of P2X7R is the promotion of effector functions in monocytes/macrophages (cell proliferation, killing, nuclear factor-kB activation, cytokine production, and release; Rossi et al., 2012) resulting in an amplification of the innate immune response, increasing recent evidence suggests that this receptor may also be actively involved in wound healing by stimulating reparation and fibrosis. Indeed, several studies have demonstrated that the P2X7R is expressed by a wide range of fibroblastic cells throughout the body, both in animals [heart (Kumagai et al., 2013), kidney (Ponnusamy et al., 2011), pancreas (Haanes et al., 2012), endometrium (Koshi et al., 2005)] and in humans [skin (Solini et al., 1999), synovium (Caporali et al., 2008), gingival, and dental pulp tissue (Jiang et al., 2015)]. In addition, increasing data from animal models indicate that P2X7R activation plays a key role in different forms of experimental fibrotic diseases. Gonçalves et al. (2006) demonstrated that kidneys from P2X7R-knockout (KO) mice with unilateral ureteral obstruction showed a lower immunostaining for transforming growth factor-β (TGF-β), and myofibroblasts when compared with wild-type mice. Moreover, P2X7R-KO mice airway-administered with bleomycin to induce lung fibrosis, presented dramatically reduced fibrosis markers, such as lung collagen content and matrix-remodeling proteins, than in control animals normally expressing the receptor (Riteau et al., 2010). More recently, beneficial effects of P2X7R antagonism on liver fibrosis were reported both in rats with bile duct ligation-induced cirrhosis, (Tung et al., 2015) and in mice with carbon tetrachloride-induced liver fibrosis (Huang et al., 2014). Keeping this in mind, we characterized the expression and function profile of the P2X7R in dermal fibroblasts from patients with SSc, a systemic fibrotic disorder of unknown origin, to test the hypothesis that a dysregulation of this receptor may play a role in this disease.

A first relevant finding from our study is the demonstration that in SSc dermal fibroblasts the surface expression of the P2X7R is substantially increased. In fact, both the percentage of P2X7-positive cells and the mean number of receptors expressed on the cell membrane, as indicated by the MFI values, was significantly higher in fibroblasts from SSc patients than healthy controls. These data confirm and expand preliminary data from a seminal work from the group of Di Virgilio (Lo Monaco et al., 2007), in which for the first time evidence was provided that SSc dermal fibroblasts expressed mRNA for several P2 receptors, including the P2X7R; however, these authors did not perform any quantitative comparison with control cells. Indeed, in our study we found no significant difference in fibroblast P2X7R-mRNA content between SSc patients and healthy subjects, a datum suggesting that P2X7R up-regulation does not involve gene transcription but only cell surface expression of the molecule, possibly via an increased trafficking of the P2X7R protein to the plasma membrane. Indirect support to this view is provided by previous data on monocytes/macrophages. Gudipaty et al. (2001) demonstrated that during cytokine-driven differentiation in macrophages healthy human monocytes markedly increase the P2X7R number per cell (MFI > 10-fold) without any significant change in the mRNA content. Moreover, we found that monocytes from patients with a chronic inflammatory disorder such as Behçet's disease expressed significantly higher P2X7R membrane levels than healthy controls, with no difference in intracellular mRNA concentration (Castrichini et al., 2014). Notably, Gu et al. (2000) demonstrated that in monocytes from healthy humans most of P2X7R protein is localized inside the cell roughly an order of magnitude greater than that expressed on the membrane. On this basis, the authors hypothesized that this intracellular pool represents a reserve able to be recruited to the surface when the cell activates. Probably such a mechanism is operative also in SSc fibroblasts, thus explaining the high cell membrane P2X7R expression found in these patients.

Not only the expression, but also the function of the P2X7R as evaluated by agonist-induced Ca^2+^ influx, αSMA expression, cell migration, CTGF, and collagen release, was enhanced in SSc dermal fibroblasts.

As regards the P2X7R-dependent Ca^2+^ influx, a significantly higher ion permeability was observed in SSc patients when compared to healthy controls. In particular, SSc fibroblasts showed an enhanced reactivity to BzATP, a potent and selective synthetic P2X7R agonist, as demonstrated by the higher percentage of responsive cells, and the increased total amount of Ca^2+^ entry into the single cell in the time unit, as reflected by AUC 5′-values. Such results, together with the evidence that SSc fibroblast hyper-responsiveness significantly reduced after oATP pre-incubation, strongly support the active role of the P2X7R in the function of these cells.

Notably, these findings paralleled the differences in the expression pattern observed between the two groups, i.e., SSc fibroblasts expressed higher levels of P2X7R on a higher number of cells when compared to controls, thus suggesting that P2X7R overexpression is the main driver of receptor overactivity. In agreement with our data, a preliminary observation by Lo Monaco et al. (2007) found that SSc dermal fibroblasts showed an increased sensitivity to extracellular ATP than healthy controls in terms of intracellular Ca^2+^ changes. Moreover, some recent studies reported that P2X7R function was increased in peripheral blood mononuclear cells (PBMC) from patients with other autoimmune diseases, specifically rheumatoid arthritis (Al-Shukaili et al., 2008, 2011), and Behçet's disease (Castrichini et al., 2014). In particular, Al-Shukaili et al. (Al-Shukaili et al., 2008, 2011) demonstrated that in rheumatoid arthritis patients the increase in the P2X7R function, without any concomitant membrane expression enhancement, was associated to a higher prevalence of a gain-of-function receptor gene polymorphism in these subjects. Conversely, our data are poorly consistent with the hypothesis that SSc-associated changes in the receptor function are the result of a specific genetic substrate. In fact, currently known gain of function polymorphisms influence receptor properties without any appreciable significant effect on the membrane expression (Sun et al., 2010). Nevertheless, a novel single-nucleotide polymorphism (H155Y) enhancing P2X7R function via increased protein trafficking and surface expression has been recently identified (Bradley et al., 2011). Thus, this theoretical possibility cannot be completely ruled out.

Exaggerated fibroblast activation, as reflected by increased cell proliferation, migration, production of ECM proteins and collagen, represents a key feature in the pathogenesis of SSc (Allanore et al., 2015). Our data provide for the first time evidence that P2X7R may be actively involved in promoting this process. In fact, SSc fibroblast stimulation with selective receptor agonists resulted in a clear-cut increase in the myofibroblast phenotype, with higher propensity of these cells in releasing collagen and migrating to close wound scratch, and these effects were completely reversed by P2X7R antagonists pre-incubation. Notably, such changes took place only when receptor activation occurred in the setting of a cell priming with LPS. This datum first suggested that inflammatory molecules could represent crucial mediators in the genesis of the phenomenon. Indeed, it is well-recognized that in monocytes LPS-induced release of inflammatory cytokines, particularly IL-1β and IL-6, is markedly boosted by P2X7R activation (Di Virgilio, 2007; Castrichini et al., 2014; Gicquel et al., 2015). Moreover, both these molecules are produced by dermal fibroblasts upon LPS stimulation, (Tardif et al., 2004; Wang et al., 2011) and are able to induce a fibrogenic phenotype in these cells (Goldring and Krane, 1987; O'Reilly et al., 2014). Despite such premises, our data conflict with this interpretative hypothesis: LPS+BzATP incubation of SSc fibroblasts did not produce any change in IL-1β release, while IL-6, although reaching significantly higher levels with respect to basal conditions (but still quite low in absolute), did not show any appreciable impact on collagen production. In fact, reproducing the *in vivo* conditions by adding the sIL-6R to cell cultures in order to make possible IL-6-dependent transsignaling pathway activation, no any further increase in PIP levels was detected. As regards the apparently unexpected IL-1β findings, it should be noted that previous studies demonstrated that IL-1α, rather than IL-1β, peculiarly plays an important role in promoting the activation of SSc dermal fibroblasts (Kawaguchi, 1994; Higgins et al., 1999; Hutyrová et al., 2004; Kawaguchi et al., 2004). In fact, while it has been reported that IL-1α is expressed in these cells where induced a fibrogenic phenotype via an autocrine mechanism (Kawaguchi et al., 2004), IL-1β was not detectable in both SSc fibroblast cell extracts and the supernatants (Kawaguchi, 1994).

An alternative intriguing mechanism by which the P2X7R could promote the development of a fibrogenic phenotype in fibroblasts may be the direct activation of intracellular pathways controlling collagen biosynthetic machinery, particularly the mitogen-activated protein kinase (MAPK) cascade. In fact, increasing evidence indicates that a key role in fibroblast activation is also played by ERK1/2, a member of the MAPK family (Mu et al., 2012). Specifically, it has been demonstrated that in SSc fibroblasts ERK1/2 activation contributes to increase the expression of fibrotic proteins, including collagen, as well as to the contraction of these cells (Bogatkevich et al., 2007; Chen et al., 2008; Lazzerini et al., 2012).

Several data suggest that the P2X7R can activate the ERK1/2 pathway in different cell types, although the mechanism of this coupling is still unclear (Amstrup and Novak, 2003; Gendron et al., 2003). Intriguingly, in addition of being activated by an increase in [Ca^2+^]_*i*_ (Gendron et al., 2003), it has been demonstrated that a sequence of the carboxy-end of the P2X7R contains a conserved LPS-binding domain (Coddou et al., 2011), and peptides derived from this sequence bind LPS *in vitro* and neutralize the ability of LPS to activate ERK1/2 in tissue cultured macrophages (Denlinger et al., 2001). Keeping this in mind, the carboxy-terminal domain of P2X7R may directly coordinate key signal transduction events related to LPS action also in SSc fibroblasts, specifically the ERK1/2 activation, eventually leading to the development of a pro-fibrotic phenotype. Our data support this point of view. Indeed, we demonstrated that LPS priming is required for P2X7R-induced collagen production from SSc cells, and that such an effect was completely abrogated when cultures were pre-incubated with a ERK1/2 inhibitor. The concomitant finding here provided that also CTGF release was induced by LPS+BzATP, and then fully reversed by both P2X7R antagonists and ERK1/2 inhibitors, points to MAPK-dependent CTGF production as a key event in promoting the P2X7R-induced fibrogenic phenotype in dermal fibroblasts. This hypothesis is supported by the evidence provided by Leask et al. (2003) that MAPK pathway activation can induce the CTGF promoter in human dermal fibroblasts.

Finally, we cannot ruled out that also indirect effects can contribute to the observed P2X7R-induced ERK1/2 signaling activation and profibrotic response. In fact, it has been demonstrated that P2X7R activation causes ATP release (Pellegatti et al., 2005; Brandao-Burch et al., 2012), which in turn can activate profibrotic P2Y2 and/or A2A receptors (in this case indirectly, by increasing extracellular adenosine concentrations), both exerting their effects through a MAPK signaling pathway (Pellegatti et al., 2011; Lazzerini et al., 2012; Lu et al., 2012).

In conclusion, our data provide for the first time evidence that in dermal fibroblasts from SSc patients both the expression and function of the P2X7R are increased with respect to healthy controls. In these cells, P2X7R stimulation induces a fibrogenic phenotype characterized by increased migration and collagen production, via activation of the ERK1/2 pathway. These findings, besides giving new insights on the pathophysiology of SSc-associated dermal fibrosis, rise the distinct possibility that drugs specifically blocking the P2X7R could represent a novel and attractive pharmacological target for anti-fibrogenetic therapy of the disease. Indeed, novel P2X7R inhibitors are available for clinical use [e.g., periodate-oxidized ATP, (Ferrero, 2009) CE-224,535, (Stock et al., 2012) and AZD9056 (Eser et al., 2015)]. Thus, P2X7R targeting may have translational potential with a possible relevant impact in morbidity and mortality in SSc patients.

## Ethics statement

Local Ethical Committee (Comitato Etico Regione Toscana—Area Vasta Sud Est) approved the study, and patients gave their oral and written informed consent in accordance with the Principles of the Declaration of Helsinki.

## Author contributions

Conception and design of the work: PL, FL, PC. Substantial contributions to the acquisition of data for the work: DG, AG, ES, FV, AA, PT, MC, VD. Substantial contributions to the analysis of data for the work: DG, PL, AG, MN, ES, FL, PC. Substantial contributions to the interpretation of data for the work: PL, AG, MN, ES, RF, FL, PC. Drafting the work: DG, PL, AG, PC. Revising the draft of the work critically for important intellectual content: DG, PL, AG, SD, DA, MM, RF, FL, PC. Final approval of the version to be published: DG, PL, AG, MN, ES, FV, AA, PT, SD, MC, VD, DA, MM, RF, FL, PC. Agreement to be accountable for all aspects of the work in ensuring that questions related to the accuracy or integrity of any part of the work are appropriately investigated and resolved: DG, PL, AG, MN, ES, FV, AA, PT, SD, MC, VD, DA, MM, RF, FL, PL.

### Conflict of interest statement

The authors declare that the research was conducted in the absence of any commercial or financial relationships that could be construed as a potential conflict of interest.
