# Supplementary material for: Searching Novel Therapeutic Targets for Scleroderma: P2X7-Receptor Is Up-regulated and Promotes a Fibrogenic Phenotype in Systemic Sclerosis Fibroblasts
Source: Front Pharmacol. 2017 Sep 13;8:638. doi: 10.3389/fphar.2017.00638 (PMC5602350; doi:10.3389/fphar.2017.00638)
Supplement: Supplementary file 1 [file Table1.docx]

**SUPPLEMENTARY TABLE.** Demography and clinical characteristics of systemic sclerosis (SSc) patients^a^ and healthy controls (HC).

**SSc HC**

Subjects, n 9 8

Female sex, n 9 8

Age, median (range), years 60 (45-80) 61 (35-76)

Disease duration^b^, median (range), years 10 (4-15) -

Disease subset, limited/diffuse, n 9/0 -

Anti-Scl-70 positive/negative, n 0/9 -

Treatment, n

Prostacyclin analogues 4 -

Endothelin-1 receptor antagonists 2 -

Calcium-channel blockers 3 -

Steroids 5 -

^a^SSc was determined according to the Subcommittee for Scleroderma Criteria of the American Rheumatism Association Diagnostic and Therapeutic Criteria Committee (Masi, 1980); ^b^from the first non-Raynaud’s manifestation.

**Reference:**

Masi, A. T. (1980). Preliminary criteria for the classification of systemic sclerosis (scleroderma). Subcommittee For Scleroderma Criteria of the American Rheumatism Association Diagnostic and Therapeutic Criteria Committee. [*Arthritis Rheum*.](https://www.ncbi.nlm.nih.gov/pubmed/7378088) 23, 581–590. doi: 10.1002/art.1780230510
