# Supplementary material for: Searching Novel Therapeutic Targets for Scleroderma: P2X7-Receptor Is Up-regulated and Promotes a Fibrogenic Phenotype in Systemic Sclerosis Fibroblasts
Source: Front Pharmacol. 2017 Sep 13;8:638. doi: 10.3389/fphar.2017.00638 (PMC5602350; doi:10.3389/fphar.2017.00638)
Supplement: Supplementary file 2 [file DataSheet1.DOC]

**SUPPLEMENTARY METHODS**

**Analysis of P2X7 mRNA by qRT-PCR**

The sequence for each reference gene was designed with Primer Express Version 2.0 (Applied Biosystems) and synthesized by Sigma-Aldrich; whenever possible, intron-spanning primers were selected to avoid amplification of genomic DNA. Reaction conditions for reference gene primer were optimized; i.e., a gradient PCR was conducted to assess the optimal annealing temperature, while a standard curve obtained by scalar dilution of a cDNA pool (1:5, 1:25, 1:125, 1:625) was always generated to verify PCR efficiency (optimal value obtained in the range of 95-105% and a linear standard curve, R2, greater than │0.990│). For Quantitect Primer assays, manufacturer’s Real-Time conditions were followed, even though a gradient Real-Time PCR was conducted to confirm the optimal annealing temperature and a dilution curve to verify the effective efficiency. P2X7 mRNA transcriptomic profile was assessed by Real-Time PCR studies. The reactions were performed in duplicate in the Bio-Rad C1000™ thermal cycler (CFX-96 Real-Time PCR detection systems, Bio-Rad Laboratories Inc., Hercules, CA, USA). For monitoring cDNA amplification Syber green was used (QuantiFast SYBR Green SuperMix). PCR was performed in a volume of 25 μl per reaction; to minimize the influence of PCR inhibitors in Real-Time applications, all cDNA samples were diluted 1:5. Reaction mixture included 2μl of template cDNA [10 ng/μL], 1 μM of each primer, 2X QuantiFast SYBR Green SuperMix and sterile H2O. Amplification protocol started with 95°C for 3 minutes followed by 39 cycles at 95°C for 10 seconds and 60°C for 30 seconds. To assess product specificity, amplicons were checked by melting curve analysis. Melting curves were generated from 65°C to 95°C with increments of 0.3°C/cycle.

The geometric mean of the three most stably expressed genes in fibroblast of SSc patients and healthy controls (eEF1a, RPL13a, RPS4X) was used for normalization of Real-time PCR results. Relative quantification of P2X7 was calculated by the ΔΔCt method using Bio-Rad’s CFX96 manager software.

**P2X7R-induced calcium influx measurement**

For single-cell [Ca^2+^]_i_ experiments, fibroblasts were plated on 8-well tissue culture chambers (Sarsted) at the density 2 × 10^5^ per well, in starvation medium (DMEM plus 0.5% FBS). The next day, after aspiration to remove non adherent cells, fibroblasts were loaded with the cell-permeant fluorescent calcium dye Fura-2 acetoxymethyl ester (AM; 3 μM; Molecular Probes, Thermo Fisher) for 20 min at 37°C. Calcium measurements were carried out at room temperature.

Fura-2-loaded cells were placed on a fluorescence image microscope and perfused with a medium containing 140 mM NaCl, 5.4 mM KCl, 1 mM MgCl_2_, 1 mM CaCl_2_, 15 mM 4-(2-hydroxyethyl)-1-piperazineethanesulfonic acid (HEPES) buffer (pH 7.4) and stimulated by the addition of BzATP (0.1 mM) followed by ATP (1 mM). For experiments performed in the absence of extracellular calcium, the medium containing 0.5 mM ethylene glycol tetra acetic acid (EGTA) with no added Ca^2+^ was used. The digital fluorescence-imaging microscopy system was mounted on a Nikon Diaphot 300 (Nikon, Tokyo, Japan) inverted microscope. Fluorescence images were collected through a Nikon oil immersion 40X/1.3 numerical aperture objective and acquired by a cooled charge-coupled device camera (Photometrics, Roper Scientific, USA) and a MetaFluor imaging system (Universal Imaging, Downingtown, PA, USA).

**Collagen supernatant assay**

96-well plates pre-coated with monoclonal antibody for PIP were incubated with 100 µl of samples, standard and peroxidase (POD)-labelled with anti-PIP antibody for 3 h at 37°C. Then washed 4 times with PBS and incubated with substrate solution (H_2_O_2_ and tetramethylbenzidine) for 15 min at RT. The reaction between POD and substrate results in color development with intensity proportional to the amount of PIP present in the samples and standards. The amount of PIP was quantified by measuring absorbance using a microplate reader (Microplate 50 Pc, BioRad Laboratories, Hercules, CA). Accurate sample concentration of the PIP was determined by comparing their specific absorbance with those obtained for standards plotted on a standard curve at 450 nm.
